# Domain and intensity of physical activity are associated with metabolic syndrome: A population-based study

**DOI:** 10.1371/journal.pone.0219798

**Published:** 2019-07-17

**Authors:** José A. Serrano-Sánchez, María Jesús Fernández-Rodríguez, Joaquin Sanchis-Moysi, María del Cristo Rodríguez-Pérez, Itahisa Marcelino-Rodríguez, Antonio Cabrera de León

**Affiliations:** 1 Research Institute of Biomedical and Health Sciences (IUIBS), Las Palmas de Gran Canaria, Canary Islands, Spain; 2 Department of Physical Education, University of Las Palmas de Gran Canaria, Las Palmas de Gran Canaria, Spain; 3 Clinical Analysis Department, Dr. Negrín University Hospital, Las Palmas, Spain; 4 Research Unit, Nuestra Señora de la Candelaria University Hospital, Santa Cruz de Tenerife, Spain; 5 Area of Preventive Medicine, University of La Laguna, La Laguna, Spain; La Inmaculada Teacher Training Centre (University of Granada), SPAIN

## Abstract

**Introduction:**

Little is known on how the domain and intensity of physical activity (PA) associates with metabolic syndrome (MetS). The aim of this study was to examine associations between PA domains (leisure-time, domestic, active transport, total walking and total PA), PA intensities (light, moderate and vigorous) and PA levels with MetS in the general adult population.

**Methods:**

Cross-sectional study. Anthropometry, blood biochemistry, 79-item PA-questionnaire, lifestyle and medical history were evaluated in a representative sample of Canary Island adults (n = 6,729). MetS was diagnosed using the harmonized IDF-NHLBI-AHA criteria. T-test and multivariable logistic regression was used to analyse associations between PA domains and intensities with MetS *vs*. no MetS, controlling for socio-demographic, lifestyle, family antecedents and body mass index (BMI).

**Results:**

For each MET-h/day spent in moderate-vigorous PA intensities, as well as in recreational domain, active transport, total walking and total PA, the odds of MetS decreased between 3–10%. Energy expenditure exclusively in light and domestic PAs was not associated with MetS, however it was important to achieve a total PA level of 3 MET-h/day, which reduced the odds of MetS by 23%. This reduction was blunted in those with more than 2 h/d of TV watching time. A PA level of 3 MET-h/d also nullified the risk of MetS in those with low PA and high TV consumption.

**Conclusions:**

Some types of leisure time PAs may contribute more than others to reducing MetS. Light and domestic PA play a complementary role in enhancing energy expenditure in the general population. TV watching time above 2 h/d counteracted the MetS risk reduction associated with PA level, but PA level also reduced the risk of METs presented by those with a low level of PA and an excess TV watching time. Physical activity explains a greater amount of the variance of MetS than any other factors of lifestyle, education, sex and family history, and substantially mitigates the strong association of age and BMI with MetS.

## Introduction

Metabolic syndrome (MetS) is a high prevalent cluster of cardio-metabolic risk factors affecting a third or more of the adult population in many countries [1,2]. MetS has been characterized as a combination of central obesity, dysglycaemia, raised blood pressure, and dyslipidaemia [3]. It represents an economic expense of thousands of millions of euros for many public health systems, due to the increase in healthcare management of cardiovascular events and type 2 diabetes [4,5], which are the immediate health risk derived from MetS [6,7]. Insulin resistance and the inflammatory state of adipose tissue have been suggested as a central mechanism in the pathophysiology of MetS [8,9]. The causes responsible for the MetS appear to be multifactorial, including lifestyle and family history [10].

Regular, moderate and vigorous physical activity (PA) has been shown as an effective lifestyle factor to prevent MetS [11,12,13]. Less is known about the relationship between light-intensity PA and MetS. The accumulation of energy expenditure in light-intensity PAs (typically < 45% VO_2_ max) [14] could play a similar role in the prevention of MetS and diabetes to the beneficial role played by moderate-vigorous PA. Light PA usually occupies a substantial proportion of the daily energy expenditure [15] and it is probably the main source of energy expenditure in the general population.

Also, little is known about the relationship between different PA domains in regard to MetS, particularly domestic PA (housework, yard work, chores). Introducing light PA breaks in sitting time blunts the negative and independent effects of prolonged sitting time on metabolic and cardiovascular health [16,17]. Thus, light-intensity and domestic-domain PAs could play a double role in the prevention of MetS, both breaking up the sedentary time and eliciting energy expenditure in a similar way to moderate and vigorous PAs, although with less time-efficiency. Some cross-sectional studies with accelerometers have reported a moderate association of light PA with MetS [18], but these studies did not provide information on PA domains, nor distinguish the activity of walking from light PA. To our knowledge, previous studies have not undertaken a comprehensive evaluation including intensities, domains and PA levels.

Thus, the aim of the present study was to examine the associations of leisure time PA according to specific domains (leisure-time, domestic, active transport, total walking across all domains and total PA), intensity (light, moderate and vigorous) and PA level with MetS in the general adult population, considering a broad set of factors (socio-demographic, lifestyle, family history and BMI). We hypothesized a gradual and inverse relationship between the energy expenditure in all PA domains and intensities with MetS in the general adult population. A secondary aim was to examine the relative contribution of the selected factors on the variability of MetS.

## Material and methods

### Sample and design

The participants (18–75 years) in this cross-sectional study were drawn from the "CDC de Canarias" cohort (Cardiovascular, Diabetes and Cancer, the name of the study in Spanish). The methodology has been described in detail elsewhere [19]. The participants were recruited randomly from the health card census, which includes almost all of the population living in the archipelago. Multi-stage stratification, with the island as the first stage and the region (north and south in each island) as the second, randomly selecting at least one municipality per region, and finally carrying out a simple random sampling in each municipality was applied. The recruitment strategy began with the support of the health authorities of each island, and the Primary Care teams that attended the selected population. The study was disseminated in the local media, and then a first mailing was sent to the selected subjects with information about the objectives of the project, and asking for their participation. Fifteen days later, a second mailing was carried out. The selected participants were invited to the health center closest to their home in a fasted state. Upon arrival, a nurse performed a physical examination and took a blood sample. Then, a second visit was scheduled to answer the CDC questionnaire. The only exclusion criterion was the inability to respond to the interviewer and not having a family member who could take over. Recruitment was completed in December 2005, obtaining a final sample size of 6,729 participants, with a response rate of 70%. The study was approved by the Bioethics Committee of Nuestra Señora de la Candelaria University Hospital, and all participants provided their informed consent in writing.

#### Measures

Each participant underwent a physical examination during which anthropometric indices were recorded. For the current analysis, we used the parameters of waist circumference (in cm), and body mass index (BMI, in kg/m^2^). Heart rate and blood pressure (mmHg) were also measured following standardized protocols. A sample of venous blood was drawn in the morning after an overnight fast of at least 10 hours. Lipid levels were measured with a Hitachi 917 autoanalyzer, and the results were recorded as the serum concentration in mg/dL. When the serum concentration of triglycerides was < 300 mg/dL, the level of low-density lipoprotein cholesterol (LDL-C) was calculated using the Friedewald formula. The level of non-high-density lipoprotein (Non-HDL-C = total cholesterol minus HDL-C) was calculated to compensate for the inaccuracy of the Friedewald equation in calculating LDL-C when triglycerides are elevated [20].

The participants were also interviewed by nurses about any relevant health-related family history, lifestyle, socio-demographics, dietary habits and PA (the instrument is available at http://www.cdcdecanarias.org). The interviewers received specific training to avoid influencing the responses and minimise a potential bias of social desirability that could cause an overestimation of PA.

#### Metabolic syndrome

Diagnosis of MetS was based on the harmonized definition of IDF-NHLBI-AHA, which classifies the participants as having MetS if they have at least 3 of the following 5 risk factors: **1)** waist circumference (WC) ≥102 cm (men) or ≥88 cm (women); **2)** triglycerides (TG) ≥150 mg/dL or taking medication to lower TG; **3)** HDL-C <40 mg/dL (men) or <50 mg/dL (women), or taking medication to improve HDL-C; **4)** systolic blood pressure ≥130 mm-Hg or diastolic blood pressure ≥85 mm-Hg or taking antihypertensive medication; or **5)** fasting glucose ≥100 mg/dL or taking medication to lower blood glucose [3].

Participants with evidence of diabetes and vascular accidents in addition to MetS were excluded from the study (n = 652, 10.1%). We followed the recommendations from the WHO consultation expert considering MetS as a premorbid condition [21]. By excluding patients with diabetes and CVD, potential sources of confusion due to the associations of diabetes and cardiovascular events with PA can be controlled [22,23]. A continuous metabolic risk index was also calculated by adding the number of positive MetS components accumulated in each participant to generate an ordinal variable with a scale from 0 to 5 positive components of MetS.

#### Physical activity

Data on PA was recorded with the Minnesota Leisure Time Physical Activity Questionnaire (MLTPAQ), which has been validated specifically in the Spanish population against cardio-respiratory fitness, showing acceptable values for the estimation of energy expenditure in PAs during the previous year (r = 0.57 in men and 0.39 in women) [24]. MLTPA asked for specific PAs in leisure-time, active transport and domestic environments, excluding work-related PAs. During the first phase of the study we expanded the 67 original items of the MLTPAQ to 78 items. New PAs belonging to the domestic-domain and of light -intensity (< 3 MET), such as taking care of people and animals, fishing shellfish, and other miscellaneous activities [25] were added to the MLTPAQ. Moderate-intensity (≥ 3 MET), domestic PAs such as, wood loading, milking, masonry and farming (see S1 Table) were also included in the MLTPAQ. Globally, the added light and moderate PAs represented 0.9 and 1.7% of the total PAs recorded. The contribution of new PAs to the light-intensity energy expenditure was less than 1% and had a negligible impact on total energy expenditure, making it unnecessary to undertake a new validation of the MLTPAQ. The new questions expanded the ability of the questionnaire to detect PAs specific to our population.

Each PA reported by the participants was assigned a metabolic equivalent task (MET) score which expresses its intensity based on the ratio between the metabolic rate during activity and the basal metabolic rate. One MET reflects an individual’s energy consumption at rest, equivalent to approximately 1 kcal (4.184 kJ) per kg body weight per hour [25].

All PAs reported were classified according to its domain as leisure (sports, exercise and recreational activities), domestic (housework, yard work, chores) and active transport (walking or bicycling for the purposes of going somewhere), and its intensity, as light (< 3.0 MET), moderate (≥ 3.0 and ≤ 6 MET) and vigorous (> 6 MET). The energy expenditure of light, moderate and vigorous PAs was calculated separately, as well as in leisure, domestic, active transport, total walking and total PA (8 PA items), following the formula *energy expenditure* = *intensity* (MET) x *weekly frequency* x *duration* (average time spent in each session in hours and minutes). Energy expenditure was expressed in MET-hours/day (MET-h/day). Participants were also classified as achieving the PA guidelines (intensity ≥ 3.5 MET, frequency ≥ 5 days/week and duration ≥ 30 min/day). Depending on their total PA, participants were also divided into < 3 MET-h/day and ≥ 3 MET-h/day categories.

#### Lifestyle

Lifestyle variables included information about: 1) night-time sleep (categorized as < 6 h/day and ≥ 6 h/day), 2) daytime napping sleep (yes or no), 3) television viewing (5 groups categorized as < 1 h/d, ≥ 1 to < 2 h/d, ≥ 2 to < 3 h/d, ≥ 3 to < 4 h/d and ≥ 4 h/d), 4) alcohol consumption (low [<40 g/day for men and <24 g/day for women] and high [≥40 g/day for men, ≥24 g/day for women], 5) current smoking (yes or no), and 6) adherence to Mediterranean diet (continuous scale from 0–10) described below.

Mediaterranean diet were evaluated using a frequency questionnaire validated for our study population [26], previously adapted from the scale proposed by Trichopoulou et al. [27]. Briefly, we considered the following food groups: cereals, fruits and nuts, vegetables, potatoes and legumes, olive oil, fish, dairy products, meat, sugar and sweets and alcoholic beverages. A value of 0 or 1 was assigned to each of the 10 indicated components using the sex-specific median as the cut-off value, assigning a value of 0 or 1 for the beneficial components below and above the median, respectively. The opposite approach was used for detrimental components of the diet, i.e. when the score was below the median a value of 1 was assigned and *vice versa*. Therefore, the total adapted score ranged from 0 (minimal adherence to the traditional Mediterranean diet) to 10 (maximal adherence).

#### Socio-demographic variables

The socio-demographic variables included were: 1) age (18–30, 31–45, 46–60 and 60–75), 2) sex (men and women), 3) education (primary or lower, secondary and university), 4) marital status (single, married/couple and separated/widow), 5) occupational activity (worker, student, unemployed, house keeper, retired and others), 6) Socio-economic status (continuous variable with a scale from 4–24 points as the result of combining family income, education and over-crowded home conditions) [28] and 7) ancestry when both parents and grandparents were born in the Canary Islands (yes or no). This variable was included to control the survival of an aboriginal genetic load among 23–38% of the Canary Islands population [29].

#### Family history

Family medical history regarding diabetes, arterial hypertension and cardiovascular diseases was recorded. In order to maintain the parsimony of the multivariate analysis, the six variables of family morbidity were categorized as a single variable with four categories: none, at least one morbidity from the father, the mother and both parents. This new variable explained a similar proportion of the variance of MetS as the six binary variables (R^2^ = 0.018 vs. 0.027), with a better goodness-of-fit.

### Data analysis

Physical activity variables were tested for normality due to a skewed distribution and transformed into z scores to test mean differences between participants with MetS *vs*. no MetS. We used a chi-square test and t-test to compare proportions and means between both groups of participants to detect uncorrelated variables with MetS. A Bonferroni post-hoc test was used to test for differences between three or more categories. Pearson and Rho Spearman correlations were applied to detect potential associations between PA domains, intensities and covariables, as appropriate. We found moderate to high correlations (0.57 to 0.76, p < 0.001) between energy expenditure in several PA domains and intensities (see S2 Table). Thus, five PA submodels were delineated for subsequent and separated multivariate analyses: 1) domains (leisure, domestic and transport), 2) intensities (light, moderate and vigorous), 3) total PA, 4) total walking across all domains, and 5) PA levels (meeting PA guidelines and ≥ 3 MET-h/day in total PA).

The associations of MetS with selected PAs was analysed with binomial logistic regression. Odds ratios (OR) and 95% confidence intervals (95% CI) for having MetS *vs*. no MetS were calculated separately for all PA variables (introduced as continuous variables in MET-h/day) and indicated covariates (introduced as described in their correspondent sections). The alpha error was set as P < 0.05 (two-sided tests). The Generalized Inflation Variance Factor (GVIF) was calculated to detect collinearity between covariates as has been previously described [30]. Some variables associated with MetS in bivariate analyses were excluded from the final model. This was the case for socio-economic status, which was derived from education, increasing GVIF and introducing inconsistency in ORs if analysed conjointly with education. The occupational activity and marital status variables increased GVIF due to association with sex and age, and their exclusion had no impact on the goodness-of-fit and predictive power in multivariate analyses. One occupational group (housekeepers *vs*. the rest) was consistent in the analyses (P < 0.05) and remained in the final model. The adherence to the Mediterranean diet was not associated in bivariate and multivariate analyses with MetS and excluded from the final model.

Since sitting time and light-intensity energy expenditure are inversely correlated (r = -0.88 to -0.96) [15,31,32], creating a problem of collinearity when trying to adjust light energy expenditure for sitting time [31,32]. Thus, in our study we used total TV time as a surrogate measure of sedentary time, which was weakly correlated with light-intensity energy expenditure and has been associated with MetS and higher cardio-metabolic risk [33,34]. We also evaluated the potential combined effects of TV watching time and PA level, adding their respective regression coefficients obtained in each step of the multivariate adjustments. Given the potential interaction between TV watching time and PA level, we introduced interaction terms in the analysis. In our study, TV watching time and PA level had a null interaction (all “p” values for interaction terms between 0.14 and 0.93). Consequently, the effects of combining both variables were only additive on the log-odds scale.

We also tested some potential interactions between sex with light and domestic energy expenditure and house-keeper, but they were not included in the final model due to lack of significance (P > 0.05). Thus, the final model included: 1) one of the PA submodels, 2) socio-demographic (age, sex, education, ancestry, house-keeper), 3) lifestyle (TV watching, night-sleep, nap, smoking, alcohol intake), 4) family history, and 5) BMI. The goodness-of-fit of the multivariate model was tested with the Hosmer and Lemeshow chi-square test, showing a correct fit of the model (from 0.141 to 0.481) [35]. A Pearson goodness-of-fit test (P = 0.968) and total percentage of correctly predicted cases (74.9%) were additionally performed to corroborate the goodness-of-fit of the final model [36]. A step-by-step multivariate logistic regression was followed to assess the relative contribution of the examined cluster variables on MetS. The percentage of change in the coefficient of determination, pseudo-R^2^ Nagelkerke (R^2^_N_), was determined to quantify the relative contribution of the covariates on the variance of MetS. The R^2^_N_ satisfied the six criteria used for measures based on R^2^ to inform about the explained variance when the same sample and predictors were used [37]. Descriptive statistic and logistic regression analyses were performed with SPSS software v.19 and GVIF analyses for multicollinearity were tested with R software.

## Results

### Sample characteristics and differences between MetS groups

Table 1 shows the characteristics of the participants and the differences between MetS group *vs*. no MetS. Overall, 26.7% of women and 30% of men had MetS. The average age of the participants was 41.8 years (Table 2), with a higher representation of women (57%) and primary or lower level of education (55.8%). The recommended level of PA was met by 44% of the participants. All socio-demographic, lifestyle and family history characteristics were consistently associated with MetS, showing age and BMI the greatest differences between their categories. The most prevalent MetS component was low HDL-C (48.4%), elevated blood pressure (41.1%) and elevated WC (35.1%). The metabolic risk index also showed consistent differences across socio-demographic, lifestyle and family history characteristics.

**Table 1:** Distribution of the participants according to socio-demographic characteristic, lifestyle, metabolic syndrome components, BMI and family history of morbidity.

		Overall		No MetS (n = 4355)	MetS (n = 1702)		*p*	No of components	
		n	%	% ± 95% CI	% ± 95% CI		*χ*^2^	Mean ± 95% CI	
Overall		6057	100	71.9 ± 1.1	28.1 ± 1.1			1.7 ± 0.03	
Sex	Women	3452	57.0	73.3 ± 1.5	26.7 ± 1.5	^a^	0.004	1.6 ± 0.05	^a^
	Men	2605	43.0	70.0 ± 1.8	30.0 ± 1.8	^b^		1.8 ± 0.05	^b^
Age	18–30 years-old	1240	20.5	92.2 ± 1.5	7.8 ± 1.5	^a^	0.000	0.9 ± 0.06	^a^
	31–45 years-old	2524	41.7	78.3 ± 1.6	21.7 ± 1.6	^b^		1.5 ± 0.05	^b^
	46–60 years-old	1778	29.4	57.3 ± 2.3	42.7 ± 2.3	^c^		2.3 ± 0.06	^c^
	> 60 years-old	515	8.5	41.9 ± 4.3	58.1 ± 4.3	^d^		2.7 ± 0.11	^d^
Education	University	922	15.2	86.0 ± 2.2	14.0 ± 2.2	^a^	0.000	1.1 ± 0.08	^a^
	Secondary	1753	28.9	81.4 ± 1.8	18.6 ± 1.8	^b^		1.4 ± 0.06	^b^
	Primary or lesser	3382	55.8	63.1 ± 1.6	36.9 ± 1.6	^c^		2.0 ± 0.05	^c^
Marital status	Single	1508	24.9	86.4 ± 1.7	13.6 ± 1.7	^a^	0.000	1.1 ± 0.06	^a^
	Married, couple	4084	67.4	67.0 ± 1.4	33.0 ± 1.4	^b^		1.9 ± 0.04	^b^
	Separated, widow	462	7.6	68.2 ± 4.2	31.8 ± 4.2	^b^		1.9 ± 0.12	^b^
Occup. activity	Worker	3709	61.2	76.1 ± 1.4	23.9 ± 1.4	^a^	0.000	1.6 ± 0.04	^a^
	Student	274	4.5	91.6 ± 3.3	8.4 ± 3.3	^b^		0.8 ± 0.12	^b^
	Retired, disabled	414	6.8	56.2 ± 6.7	47.3 ± 6.7	^c^		2.3 ± 0.16	^c^
	Unemployed	457	7.5	76.1 ± 3.9	23.9 ± 3.9	^a^		1.5 ± 0.12	^a^
	House keeper	1146	18.9	57.4 ± 2.9	42.6 ± 2.9	^c^		2.2 ± 0.08	^c^
Ancestry	No	1067	17.6	75.4 ± 2.6	24.6 ± 2.6	^a^	0.005	1.6 ± 0.08	^a^
	Yes	4969	82.0	71.2 ± 1.3	28.8 ± 1.3	^b^		1.7 ± 0.04	^b^
TV watching	< 1 h/d	2067	34.1	73.9 ± 1.7	26.1 ± 1.7	^a^	0.000	1.64 ± 0.06	^a^
	1–< 2 h/d	2014	33.3	74.6 ± 1.7	25.4 ± 1.7	^a^		1.62 ± 0.06	^a^
	2–< 3 h/d	1481	24.5	70.2 ± 1.9	29.8 ± 1.9	^b^		1.75 ± 0.07	^b^
	3–< 4 h/d	369	6.1	59.9 ± 4.6	40.1 ± 4.6	^c^		2.04 ± 0.15	^c^
	≥ 4 h/d	126	2.1	50.0 ± 7.2	50.0 ± 7.2	^c^		2.36 ± 0.24	^d^
Smoker	No	4437	73.3	69.9 ± 1.3	30.1 ± 1.3	^a^	0.000	1.8 ± 0.04	^a^
	Yes	1613	26.6	77.6 ± 2.0	22.4 ± 2.0	^b^		1.5 ± 0.06	^b^
Alcohol intake	Low	5700	94.8	72.4 ± 1.2	27.6 ± 1.2	^a^	0.001	1.7 ± 0.04	^a^
	High	349	5.2	65.0 ± 5.6	35.0 ± 5.6	^b^		2.1 ± 0.15	^b^
Nap	No	4443	73.4	74.0 ± 1.3	26.0 ± 1.3	^a^	0.000	1.6 ± 0.04	^a^
	Yes	1614	26.6	66.2 ± 2.3	33.8 ± 2.3	^b^		1.9 ± 0.07	^b^
Meet PA guidelines	No	3375	55.7	69.9 ± 1.3	30.1 ± 1.3	^a^	0.000	1.8 ± 0.04	^a^
	Yes	2682	44.3	77.6 ± 2.0	22.4 ± 2.0	^b^		1.6 ± 0.07	^b^
Body mass index	Normal	2229	36.8	95.2 ± 0.9	4.8 ± 0.9	^a^	0.000	0.8 ± 0.04	^a^
	Overweight	2344	38.7	70.4 ± 1.8	29.6 ± 1.8	^b^		1.8 ± 0.05	^b^
	Obese	1479	24.4	39.3 ± 2.5	60.7 ± 2.5	^c^		2.9 ± 0.06	^c^
Family morbidity §	Neither	1751	28.9	78.0 ± 1.9	22.0 ± 1.9	^a^	0.000	1.5 ± 0.06	^a^
	Father	1260	20.8	74.6 ± 2.4	25.4 ± 2.4	^a^		1.6 ± 0.07	^b^
	Mather	1485	24.5	69.6 ± 2.3	30.4 ± 2.3	^b^		1.8 ± 0.07	^c^
	Both parents	1561	25.8	65.2 ± 2.4	34.8 ± 2.4	^b^		1.9 ± 0.07	^d^
Low HDL-C	No	3085	50.9	92.3 ± 0.9	7.7 ± 0.9	^a^	0.000	0.9 ± 0.03	^a^
	Yes	2929	48.4	50.7 ± 1.8	49.3 ± 1.8	^b^		2.5 ± 0.04	^b^
Elevated blood pressure	No	3510	57.9	92.3 ± 0.9	7.7 ± 0.9	^a^	0.000	1.0 ± 0.03	^a^
	Yes	2489	41.1	43.6 ± 1.9	56.4 ± 1.9	^b^		2.7 ± 0.05	^b^
Elevated Waist circumference	No	3872	63.9	90.1 ± 0.9	9.9 ± 0.9	^a^	0.000	1.1 ± 0.03	^a^
	Yes	2124	35.1	40.4 ± 2.1	59.6 ± 2.1	^b^		2.8 ± 0.05	^b^
Elevated Fasting Glucosa	No	4812	79.4	80.7 ± 1.1	19.3 ± 1.1	^a^	0.000	1.4 ± 0.03	^a^
	Yes	1183	19.5	38.3 ± 2.8	61.7 ± 2.8	^b^		3.0 ± 0.07	^b^
Elevated TG	No	4559	75.3	85.7 ± 1.0	14.3 ± 1.0	^a^	0.000	1.2 ± 0.03	^a^
	Yes	1483	24.5	30.1 ± 2.3	69.9 ± 2.3	^b^		3.1 ± 0.06	^b^

95% CI = 95% interval confidence;

^a,b,c,^ represent p < 0.05 for differences among row categories. Same superscript letter implies no differences between concerned categories. Different letters imply significant differences between them. Metabolic risk index is the population mean of the number of MetS components accumulated in each participant in a scale from 0 to 5.

^§^ At least one precedent of diabetes mellitus, cardiovascular disease or arterial hypertension from mother, father or both parents. HDL-C = High Density Lipoprotein Cholesterol; TG = Triglycerides; MetS = Metabolic Syndrome.

**Table 2:** Distribution of energy expenditure in physical activities, socio-demographic, lifestyle and metabolic syndrome components.

	No MetS (n = 4,355)	MetS (n = 1,702)		Overall (n = 6,057)
	Mean ± 95% CI	Mean ± 95% CI		Mean ± 95% CI
Physical activity				
Recreational EE (MET-h/day)	2.9 ± 0.10	2.0 ± 0.13	*	2.7 ± 0.08
Domestic EE (MET-h/day)	2.4 ± 0.08	3.0 ± 0.14	*	2.6 ± 0.07
Active transport EE (MET-h/day)	0.8 ± 0.03	0.7 ± 0.05	*	0.7 ± 0.03
Total walking EE (MET-h/day)	1.9 ± 0.06	1.8 ± 0.09	*	1.9 ± 0.05
Total EE (MET-h/day)	6.0 ± 0.12	5.7 ± 0.18	*	5.9 ± 0.10
Light EE (MET-h/day)	2.8 ± 0.08	3.4 ± 0.13	*	3.0 ± 0.07
Moderate EE (MET-h/day)	1.9 ± 0.08	1.4 ± 0.10	*	1.8 ± 0.07
Vigorous EE (MET-h/day)	1.3 ± 0.07	0.8 ± 0.07	*	1.2 ± 0.05
Socio-demographic and lifestyle				
Age (years)	38.9 ± 0.35	49.0 ± 0.53	*	41.8 ± 0.32
SEE score ^§^	13.9 ± 0.11	12.6 ± 0.15	*	13.5 ± 0.09
BMI (kg/m^2^)	25.5 ± 0.12	30.8 ± 0.21	*	27.0 ± 0.12
TV watching (hours/day)	1.8 ± 0.25	2.0 ± 0.53	*	1.9 ± 0.24
Alcohol intake (gr/day)	7.6 ± 0.52	9.3 ± 1.0	*	8.1 ± 0.47
Mediterranean diet score ^§§^	4.7 ± 0.05	4.8 ± 0.08		4.7 ± 0.04
Night-sleep (hours/day)	7.0 ± 0.04	6.7 ± 0.07	*	6.9 ± 0.03
MetS components				
Metabolic risk index	1.0 ± 0.02	3.5 ± 0.03	*	1.7 ± 0.03
Fasting glucose (mg/dl)	89.6 ± 0.43	97.4 ± 0.53	*	91.8 ± 0.36
Waist circumference (cm)	85.4 ± 0.34	100.4 ± 0.50	*	89.5 ± 0.33
TG (mg/dl)	94.1 ± 1.5	172.0 ± 4.64	*	115.6 ± 1.90
LDL-cholesterol (mg/dl)	120.8 ± 1.01	141.9 ± 1.82	*	126.6 ± 0.92
HDL-cholesterol (mg/dl)	54.1 ± 0.39	45.3 ± 0.58	*	51.7 ± 0.34
Total cholesterol (mg/dl)	193.7 ± 1.13	222.3 ± 2.02	*	201.6 ± 1.04
Systolic BP (mmHg)	116.6 ± 0.47	134.6 ± 0.85	*	121.5 ± 0.46
Diastolic BP (mmHg)	74.2 ± 0.30	85.1 ± 0.49	*	77.2 ± 0.29

95% CI = 95% interval confidence;

* *p* < 0.05 for differences between MetS and no MetS.

EE = energy expenditure. Metabolic risk index is the mean of the number of positive MetS components accumulated in each participant, in a scale from 0 to 5.

^**§**^ Socioeconomic status from a validated scale, combining family income, education and family overcrowding (4–24 points, Cabrera de Leon et al. 2009).

^**§§**^ Mediterranean diet score from a validated scale (0–10 points, Trichopoulou A, et al., 1995).

LDL = Low Density Lipoprotein; HDL = High Density Lipoprotein Cholesterol; TG = Triglycerides; BP = Blood Pressure; MetS = Metabolic Syndrome.

Table 2 shows the differences for continuous variables, including PA and some socio-demographic and lifestyle factors. In regard to PA domains, the participants with MetS had lower energy expenditure than those without MetS (all P < 0.05) in recreational PA (−0.9 MET-h/day), active transport (−0.1 MET-h/day), total walking (−0.1 MET-h/day) and total PA (-0.3 MET-h/day), while in the domestic-domain PA they had higher energy expenditure (+0.6 MET-h/day). In relation to PA intensities, participants with MetS showed lower energy expenditure in moderate PA (−0.5 MET-h/day) and vigorous PA (−0.5 MET-h/day) and higher energy expenditure in light PA (+0.6 MET-h/day). Also, those with MetS spent 0.2 h/day more watching TV, consumed 2.3 gr/day more alcohol and slept 0.3 h/day less than those without MetS (all P < 0.05). Compared to the no-MetS group, the MetS group was 10.1 years older and had a higher BMI (30.8 ± 0.21 kg/m^2^ *vs*. 25.5 ± 0.12 kg/m^2^, mean ± 95% CI, respectively). Socioeconomic status also showed a slight difference between both MetS groups.

### Contribution of physical activity and factors analysed in MetS

Cumulative and single R^2^_N_, and the relative percentage of contribution of the factors analysed in the variability of MetS are shown in Table 3. Total R^2^_N_ for the selected model was 0.399, corresponding to the greatest relative contribution to BMI (45.1%), followed by socio-demographic cluster variables (age, sex, education, and housekeeper with 40.9%), physical activity domains (10%), lifestyle (TV watching, night-sleep, nap, smoking and alcohol intake with 3%) and family history (1.5%). Goodness-of-fit results were acceptable in each step of the multivariate analyses.

**Table 3:** Statistic of predictive power and goodness-of-fit test in step-by-step regression analysis.

	Determination coefficient (R^2^_N_)			Goodness-of-fit		
*Order in analysis*	Cumulat.	Single	% relative	H-L test	% cases	Pearson
Physical activity	0.040	0.040	10.0	0.353	46.4	0.030
Socio-demographic	0.203	0.163	40.9	0.149	66.7	0.479
Age	0.177	0.137	34.3	0.429	54.0	-
Sex	0.182	0.005	1.3	0.065	68.2	-
Education	0.195	0.013	3.3	0.061	68.0	-
Housekeeper	0.203	0.008	2.0	0.149	66.7	-
Lifestyle	0.215	0.012	3.0	0.481	68.0	0.615
TV watching	0.208	0.005	1.3	0.356	67.4	-
Sleep	0.211	0.003	0.8	0.284	67.5	-
Nap	0.211	0.000	0.0	0.211	67.7	-
Smoker	0.215	0.003	0.8	0.391	68.0	-
Alcohol	0.215	0.000	0.0	0.481	68.0	-
Family history	0.221	0.006	1.5	0.167	67.9	0.530
BMI	0.399	0.178	45.1	0.141	74.9	0.968
Total	0.399	0.399	100	0.141	74.9	0.968

R^2^_N_ = Nagelkerke pseudo R^2^; H-L test = *p*-value of Hosmer and Lemeshow test; % cases = percentage of correctly predicted cases; Pearson = *p*-value of Pearson *χ*^2^ test.

### Associations of physical activity and factors analysed with MetS

**Fig 1:**
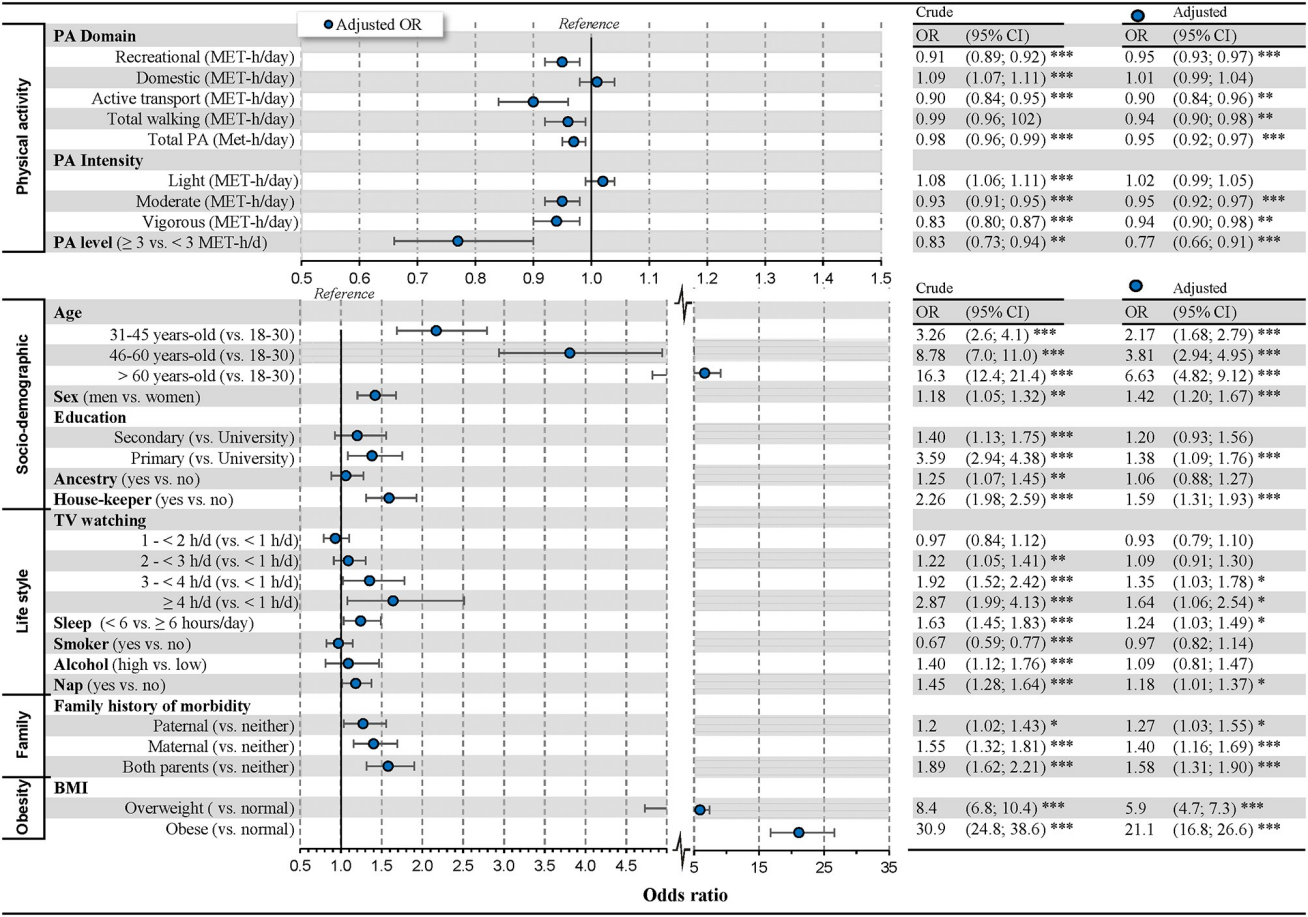
Associations of physical activity, socio-demographic, lifestyle, family history and obesity with metabolic syndrome. Note: OR = Odds ratio for having metabolic syndrome; 95% CI = 95% confidence interval for odds-ratio; * p < 0.05, ** p < 0.01, *** p < 0.001. The OR for physical activity expresses the change for each increment of 1 MET-h per day.

Fig 1 shows crude and multivariate adjusted results of logistic regression with the final selected model. After the adjustments, all PA domains, except the domestic, showed an inverse relationship with MetS. For each MET-h/day in recreational PAs, active transports, total walking and total PA, the odds of MetS decreased between 3% (OR = 0.97, P < 0.01) and 10% (OR = 0.90, P < 0.01). Domestic PA showed a positive bivariate relationship with MetS (OR = 1.09, P < 0.001), but after adjusting for the socio-demographic variables, domestic PA did not show a consistent association (see S3 Table). The types of PA intensities showed similar behaviour as PA domains: light PA lost its positive association with MetS, and moderate and vigorous PA maintained their inverse association after adjustments.

Age showed a strong bivariate association with MetS (OR between 3.26 and 16.3, p < 0.001), which was mitigated after adjusting for PA (OR between 2.57 and 9.6, p < 0.001, see S3 Table). Adding adjustment for lifestyle and BMI factors further reduced the strong bivariate association of age with MetS (OR between 2.17 and 6.63, P < 0.001, Fig 1). Those with primary education (*vs*. university) showed 38% higher odds of MetS (P < 0.05). Likewise, men (*vs*. women) and housekeepers (*vs*. the rest) showed higher odds of MetS (OR = 1.42 and OR = 1.59 respectively, all P < 0.001).

Table 4 reports the odds ratio by combining TV watching with PA level. The analyses with the combined variable (PA level & TV watching) indicate that in participants with No PA level, the ORs of MetS increases with the number of TV watching per day. Interestingly, our results also indicate that increasing the level of PA counteracts the negative effects of TV watching (Table 4).

**Table 4:** Odds ratio to have Metabolic Syndrome considering combined effects of physical activity level and TV watching.

	Crude		Adjusted	
	OR	95% CI	OR	95% CI
No PA level				
No PA level & < 1 h/d TV	1.00			
No PA level & ≥ 1 < 2 h/d TV	0.97	(0.84; 1.12)	0.93	(0.79; 1.10)
No PA level & ≥ 2 < 3 h/d TV	1.23	(1.06; 1.43) **	1.09	(0.91; 1.30)
No PA level & ≥ 3 < 4 h/d TV	1.93	(1.53; 2.44) ***	1.35	(1.03; 1.78) *
No PA level & ≥ 4 h/d TV	2.90	(2.01; 4.16) ***	1.64	(1.06; 2.54) *
PA level				
PA level & < 1 h/day TV	0.74	(0.60; 0.92) **	0.66	(0.52; 0.84) ***
PA level & ≥ 1 < 2 h/d TV	0.79	(0.65; 0.96) *	0.70	(0.56; 0.86) **
PA level & ≥ 2 < 3 h/d TV	1.01	(0.82; 1.23)	0.80	(0.64; 1.00)
PA level & ≥ 3 h/day TV	1.58	(1.21; 2.07) ***	1.12	(0.83; 1.50)
PA level & ≥ 4 h/day TV	2.37	(1.61; 3.49) ***	1.27	(0.83; 1.95)

No PA level: < 3 Met-h/d; PA level: ≥ 3 Met-h/d in total energy expenditure.

## Discussion

This study was designed to examine associations of PA domains and intensities with MetS and evaluate the relative contribution of PA in MetS. The main findings confirm the hypothesis about a gradual and inverse relationship of energy expenditure in any leisure time domain or of any intensity with MetS, with the exception of light-intensity and domestic-domain PAs. Moderate and vigorous PA intensities, as well as PA in recreational domains, active transport, total walking and total PA were associated with an independent and gradual reduction in the odds of MetS. For each MET-h/day spent in the aforementioned PAs, equivalent to between 15–20 min/day of moderate-intensity PA and between 7–10 min/day of vigorous-intensity PA, the odds of MetS decreased between 3–10%. Likewise, the level of 3 MET-h/day in total PA showed an independent and inverse relationship with MetS, reducing its odds by 23% compared to those below 3 MET-h/day.

Our results regarding moderate and vigorous PA are in line with previous research. Increasing aerobic PA (40–80% VO_2max_, 150–170 min/week, for 20 weeks to 3.2 years) has been shown to reduce MetS prevalence by 23–47% in inactive middle-age and older adults without chronic diseases [11,12], or with impaired glucose tolerance [38]. Likewise, reductions in the risk of MetS have been reported for resistance training [39] and the combination of aerobic PA with resistance exercises [12]. In our study sample, resistance exercise had a low prevalence (n = 67, 1%, S1 Table). Thus, a strategic effort in creating opportunities for and promoting PA including resistance exercises in the Canary population will likely be beneficial in reducing the prevalence of MetS.

Although no fitness data were collected in this study, our results support the existence of a minimal intensity threshold which is likely necessary to increase the VO_2_ max and elicit physiological adaptations [14]. Moderate and higher intensities activate molecular mechanisms which are important for oxidation of fatty acids and muscle glucose uptake [40,41]. In the case of MetS patients, this may be even more relevant in light of studies showing reduced responsiveness of AMP-activated protein kinase (AMPK) to exercise in obese and diabetic patients. [42,43]. Given the interaction between physical activity and fitness, and the fact that physical activity may improve MetS diagnostic factors independently of VO_2_max [44], longitudinal intervention studies would be needed to isolate the independent contribution of fitness and physical activity to MetS.

Whether the intensity of PAs or total amount of energy expended in these is more important remains controversial. There is sufficient evidence supporting that both provide beneficial effects in different health outcomes. For MetS, some prospective studies have reported greater efficiency of brisk walking compared to walking amount in reducing the progression to MetS (AHA-criteria) [45] and CVD [46]. Keeping the PA volume constant, it has been shown that interval exercise (70% VO_2max_) is better than continuous exercise (50% VO_2max_) in enhancing endothelial function and insulin signalling in fat and skeletal muscle, and reducing blood glucose and lipogenesis in adipose tissue of MetS patients (ATPIII criteria) [13]. Thus, it has been suggested that there is a greater relative importance of intensity over amount of energy expenditure for lipid control when the total amount of PA is constant or equivalent in energy expenditure [47]. In our study, both the intensity and the total amount of energy expended in PA were associated with reduced prevalence of MetS. Interestingly, our data also indicates that light-intensity PAs are not associated with lower prevalence of MetS after adjusting for moderate and vigorous PA, socio-demographic and lifestyle factors.

Contrary to our expectations, the recommended PA level in our study was not associated with the prevalence of MetS. However, those participants who supplemented some moderate-vigorous PA with light PA, until reaching a level of 3 MET-h/day in total PA, had lower odds of MetS. This PA level was equivalent to 50 minutes of moderate PA or a combination of 25–30 min/day of moderate PA plus 50–60 min/day of light PA. Even though light-intensity did not reach significance as individually related to MetS, it is likely that light-intensity PA plays a complementary role in achieving a sufficient amount of total PA to prevent MetS in the general population. For a greater benefit, a large amount of light-intensity PA should be accompanied by some time exercising at moderate or vigorous intensity. Short breaks with light PAs between prolonged sitting may contribute to reduce MetS with beneficial changes in lipid profile, blood pressure and insulin resistance [16,17].

Some cross-sectional studies with accelerometer-derived measures point towards a protective role of high levels of light PA to reduce the prevalence of MetS [31,32,48] in healthy middle-aged adults. A recent review has suggested that light PA appears to be associated with triglyceride levels and insulin, but nor with BMI, HDL-C, blood pressure and glucose levels [18]. In pre-diabetic adults, 90 min/day of light-intensity walking (2–3 km/h) produced slight beneficial effects in insulin resistance, lipid profile and visceral fat in three months [49]. Similar results have been reported in older adults who improved insulin sensitivity, triglycerides and MetS score by adding walking at low intensity compared with inactive controls [50,51]. In contrast, light-intensity PA was not related with a lower risk of developing MetS in some longitudinal studies with middle-aged and older adults [45,52]. In agreement with previous studies [53,54], domestic PA was not associated with MetS. Most of the domestic-domain PA activities in this study were of light-intensity (r = 0.74, p <0.05), including activities as standing and performing some steps and movements of arms as in cleaning, sweeping or dusting, all of them requiring low levels of oxygen consumption [14,25].

The results from the analysis combining a PA level ≥ 3 MET-h/d and TV watching revealed that a TV watching time greater than 2 h/d counteracted the benefits for MetS associated with PA level. Above 3 h/d such benefit was lost, and the OR for a MetS changed to positive although non-statistically significant. Interestingly, meeting the PA level also reduced the individual risk of TV watching for MetS, even in the participants with more than 4 h/d of TV watching in comparison with the participants with a low PA Level (Table 4).

As noted in Table 3, two characteristics that explained the most variance in the prevalence of MetS were BMI and age at 45.1% and 34.3%, respectively. PA explained 10% of the variance in the prevalence of MetS. As noted in other studies, regular PA, along with diet, have the potential to modify BMI and is important in modifying the risk for developing MetS [55].

### Limitations and strengths

One of the strengths of the present investigation was the stability of the weather in the Canary Islands (18–24°C year round temperatures, 65–70% ambient humidity and 21 days of rain per year) reducing the potential variability due to seasonal changes in walking, which is one of the most prevalent PAs in the general population. Likewise, the comparisons between domains and intensities of PA are based on measurements with the same group of participants and the same methodology.

Physical activity was assessed by questionnaire, which is less precise than other objective methods (e.g. accelerometers). Nevertheless, questionnaires are the most cost-effective methods for the assessment of PA in large populations, enabling the estimation of patterns and trends with moderate validity and good reliability [56]. In the present study, the use of a questionnaire was necessary to identify PA domains. Although sex may be a factor influencing the effects of PA on MetS, several studies indicate that the role played by sex is likely small [57,58,59]. Thus, we deliberately excluded sex a segmentation variable, to avoid excessively large confidence intervals, which would have complicated the interpretation of our results. The latter implies that our results cannot distinguish between men and women. Given the cross-sectional design, the present study does not allow the establishment of cause-and-effect relationships. We also limited the focus of our study to the previous stage of the morbid condition of MetS, excluding participants with diabetes and CVD, to enhance the specificity of the findings for primary prevention.

## Conclusions

Energy expenditure in PAs that entail walking and those of moderate and vigorous intensity, of a recreational nature and in active transport were associated with a progressive reduction of MetS for each increment of 1 MET-h/day. Energy expenditure exclusively in light and domestic PAs was not associated with MetS after adjusting for moderate and vigorous PA, TV watching and other socio-demographic characteristics, lifestyle, family history and BMI. Light PA was important to achieve a PA level of 3 MET-h/day, which was associated with 23% lower prevalence of MetS, suggesting a complementary role in enhancing energy expenditure. TV Watching time above 2 h/d counteracted the MetS risk reduction associated with PA level, but PA level also reduced the risk of MetS presented by the participants with a low level of PA and an excess of TV watching time. Physical activity explained a greater fraction of the variance for MetS prevalence compared to other lifestyle factors, education, sex and family history, and substantially mitigated the strong association of age and BMI with MetS.

## Supporting information

S1 Table (PDF)

S2 Table (PDF)

S3 Table (PDF)

S1 Database (SAV)

## References

[1] Ford ES, Li C, Zhao G (2010) Prevalence and correlates of metabolic syndrome based on a harmonious definition among adults in the US. J Diabetes 2: 180–193. 10.1111/j.1753-0407.2010.00078.x 20923483

[2] Fernandez-Berges D, Cabrera de Leon A, Sanz H, Elosua R, Guembe MJ, et al (2012) Metabolic syndrome in Spain: prevalence and coronary risk associated with harmonized definition and WHO proposal. DARIOS study. Rev Esp Cardiol 65: 241–248. 10.1016/j.recesp.2011.10.015 22305818

[3] Alberti KG, Eckel RH, Grundy SM, Zimmet PZ, Cleeman JI, et al (2009) Harmonizing the metabolic syndrome: a joint interim statement of the International Diabetes Federation Task Force on Epidemiology and Prevention; National Heart, Lung, and Blood Institute; American Heart Association; World Heart Federation; International Atherosclerosis Society; and International Association for the Study of Obesity. Circulation 120: 1640–1645. 10.1161/CIRCULATIONAHA.109.192644 19805654

[4] Scholze J, Alegria E, Ferri C, Langham S, Stevens W, et al (2010) Epidemiological and economic burden of metabolic syndrome and its consequences in patients with hypertension in Germany, Spain and Italy; a prevalence-based model. BMC Public Health 10: 529 10.1186/1471-2458-10-529 20813031 PMC2940918

[5] Marangos PJ, Okamoto LJ, Caro JJ (2010) Economic Burden of the Components of the Metabolic Syndrome In: Preedy VR, Watson RR, editors. Handbook of Disease Burdens and Quality of Life Measures. New York, NY: Springer 10.1007/978-0-387-78665-0_64

[6] Mottillo S, Filion KB, Genest J, Joseph L, Pilote L, et al (2010) The metabolic syndrome and cardiovascular risk a systematic review and meta-analysis. J Am Coll Cardiol 56: 1113–1132. 10.1016/j.jacc.2010.05.034 20863953

[7] Grundy SM (2012) Pre-diabetes, metabolic syndrome, and cardiovascular risk. J Am Coll Cardiol 59: 635–643. 10.1016/j.jacc.2011.08.080 22322078

[8] Oda E (2012) Metabolic syndrome: its history, mechanisms, and limitations. Acta Diabetologica 49: 89–95. 10.1007/s00592-011-0309-6 21720880

[9] Roberts CK, Hevener AL, Barnard RJ (2013) Metabolic syndrome and insulin resistance: underlying causes and modification by exercise training. Compr Physiol 3: 1–58. 10.1002/cphy.c110062 23720280 PMC4129661

[10] Dincer UD (2012) Cardiac ryanodine receptor in metabolic syndrome: is JTV519 (K201) future therapy? Diabetes Metab Syndr Obes 5: 89–99. 10.2147/DMSO.S30005 22563249 PMC3340112

[11] Katzmarzyk PT, Leon AS, Wilmore JH, Skinner JS, Rao DC, et al (2003) Targeting the metabolic syndrome with exercise: evidence from the HERITAGE Family Study. Med Sci Sports Exerc 35: 1703–1709. 10.1249/01.MSS.0000089337.73244.9B 14523308

[12] Stewart KJ, Bacher AC, Turner K, Lim JG, Hees PS, et al (2005) Exercise and risk factors associated with metabolic syndrome in older adults. Am J Prev Med 28: 9–18. 10.1016/j.amepre.2004.09.006 15626550

[13] Tjonna AE, Lee SJ, Rognmo O, Stolen TO, Bye A, et al (2008) Aerobic interval training versus continuous moderate exercise as a treatment for the metabolic syndrome: a pilot study. Circulation 118: 346–354. 10.1161/CIRCULATIONAHA.108.772822 18606913 PMC2777731

[14] Garber CE, Blissmer B, Deschenes MR, Franklin BA, Lamonte MJ, et al (2011) American College of Sports Medicine position stand. Quantity and quality of exercise for developing and maintaining cardiorespiratory, musculoskeletal, and neuromotor fitness in apparently healthy adults: guidance for prescribing exercise. Med Sci Sports Exerc 43: 1334–1359. 10.1249/MSS.0b013e318213fefb 21694556

[15] Healy GN, Dunstan DW, Salmon J, Cerin E, Shaw JE, et al (2007) Objectively measured light-intensity physical activity is independently associated with 2-h plasma glucose. Diabetes Care 30: 1384–1389. 10.2337/dc07-0114 17473059

[16] Peddie MC, Bone JL, Rehrer NJ, Skeaff CM, Gray AR, et al (2013) Breaking prolonged sitting reduces postprandial glycemia in healthy, normal-weight adults: a randomized crossover trial. Am J Clin Nutr 98: 358–366. 10.3945/ajcn.112.051763 23803893

[17] Thosar SS, Bielko SL, Mather KJ, Johnston JD, Wallace JP (2015) Effect of prolonged sitting and breaks in sitting time on endothelial function. Med Sci Sports Exerc 47: 843–849. 10.1249/MSS.0000000000000479 25137367

[18] Amagasa S, Machida M, Fukushima N, Kikuchi H, Takamiya T, et al (2018) Is objectively measured light-intensity physical activity associated with health outcomes after adjustment for moderate-to-vigorous physical activity in adults? A systematic review. Int J Behav Nutr Phys Act 15: 65 10.1186/s12966-018-0695-z 29986718 PMC6038338

[19] Cabrera de Leon A, Rodriguez Perez Mdel C, Almeida Gonzalez D, Dominguez Coello S, Aguirre Jaime A, et al (2008) [Presentation of the "CDC de Canarias" cohort: objectives, design and preliminary results]. Rev Esp Salud Publica 82: 519–534. 10.1590/S1135-57272008000500007. 19039505

[20] Hirsch G, Vaid N, Blumenthal RS (2002) Perspectives: The significance of measuring non-HDL-cholesterol. Prev Cardiol 5: 156–159. 10.1111/j.1520-037X.2002.00980.x. 12091759

[21] Simmons RK, Alberti KG, Gale EA, Colagiuri S, Tuomilehto J, et al (2010) The metabolic syndrome: useful concept or clinical tool? Report of a WHO Expert Consultation. Diabetologia 53: 600–605. 10.1007/s00125-009-1620-4 20012011

[22] Fan S, Chen J, Huang J, Li Y, Zhao L, et al (2015) Physical activity level and incident type 2 diabetes among Chinese adults. Med Sci Sports Exerc 47: 751–756. 10.1249/MSS.0000000000000471 25116084

[23] Phillips BE, Kelly BM, Lilja M, Gustavo Ponce-Gonzalez J, Brogan RJ, et al (2017) A Practical and Time-Efficient High-Intensity Interval Training Program Modifies Cardio-Metabolic Risk Factors in Adults with Risk Factors for Type II Diabetes. Front Endocrinol 8 10.3389/fendo.2017.00229 28943861 PMC5596071

[24] Elosua R, Marrugat J, Molina L, Pons S, Pujol E (1994) Validation of the Minnesota Leisure Time Physical Activity Questionnaire in Spanish men. The MARATHOM Investigators. Am J Epidemiol 139: 1197–1209. 10.1093/oxfordjournals.aje.a116966 8209878

[25] Ainsworth BE, Haskell WL, Herrmann SD, Meckes N, Bassett DR Jr, et al (2011) 2011 Compendium of Physical Activities: a second update of codes and MET values. Med Sci Sports Exerc 43: 1575–1581. 10.1249/MSS.0b013e31821ece12 21681120

[26] Aguirre-Jaime A, Cabrera de Leon A, Dominguez Coello S, Borges Alamo C, Carrillo Fernandez L, et al (2008) [Validation of a food intake frequency questionnaire adapted for the study and monitoring of the adult population of the Canary Islands, Spain]. Rev Esp Salud Publica 82: 509–518. 10.1590/s1135-57272008000500006. 19039504

[27] Trichopoulou A, Costacou T, Bamia C, Trichopoulos D (2003) Adherence to a Mediterranean diet and survival in a Greek population. N Engl J Med 348: 2599–2608. 10.1056/NEJMoa025039 12826634

[28] Cabrera de Leon A, Rodriguez Perez MC, Dominguez Coello S, Rodriguez Diaz C, Rodriguez Alvarez C, et al (2009) [Validation of the ICE model to assess social class in the adult population]. Rev Esp Salud Publica 83: 231–242. 19626250 10.1590/s1135-57272009000200007

[29] Maca-Meyer N, Villar J, Perez-Mendez L, Cabrera de Leon A, Flores C (2004) A tale of aborigines, conquerors and slaves: Alu insertion polymorphisms and the peopling of Canary Islands. Ann Hum Genet 68: 600–605. 10.1046/j.1529-8817.2003.00125.x 15598218

[30] Fox J, Weisberg S (2011) An R companion to applied regression, Second Edition Thousand Oaks, CA: Sage 10.1080/10543406.2012.635980

[31] Kim J, Tanabe K, Yokoyama N, Zempo H, Kuno S (2013) Objectively measured light-intensity lifestyle activity and sedentary time are independently associated with metabolic syndrome: a cross-sectional study of Japanese adults. Int J Behav Nutr Phys Act 10: 30 10.1186/1479-5868-10-30 23452372 PMC3599104

[32] Ekblom O, Ekblom-Bak E, Rosengren A, Hallsten M, Bergstrom G, et al (2015) Cardiorespiratory Fitness, Sedentary Behaviour and Physical Activity Are Independently Associated with the Metabolic Syndrome, Results from the SCAPIS Pilot Study. PLoS One 10: e0131586 10.1371/journal.pone.0131586 26120842 PMC4486454

[33] Wijndaele K, Healy GN, Dunstan DW, Barnett AG, Salmon J, et al (2010) Increased cardiometabolic risk is associated with increased TV viewing time. Med Sci Sports Exerc 42: 1511–1518. 10.1249/MSS.0b013e3181d322ac 20139784

[34] Dunstan DW, Salmon J, Owen N, Armstrong T, Zimmet PZ, et al (2004) Physical activity and television viewing in relation to risk of undiagnosed abnormal glucose metabolism in adults. Diabetes Care 27: 2603–2609. 10.2337/diacare.27.11.2603 15504993

[35] Tabatchnick BG, Fidell LS (2007) Using multivariate statistics (5th ed.). Boston: Pearson Education Inc.

[36] Allison PD. Measures of fit for logistic regression; 2014. SAS Global Forum. pp. 1–12. Retrieved from https://statisticalhorizons.com/wp-content/uploads/GOFFor%20LogisticRegression-Paper.pdf

[37] Menard S (2000) Coefficients of Determination for Multiple Logistic Regression Analysis. Am Stat 54:: 17–24. 10.2307/2685605.

[38] Orchard TJ, Temprosa M, Goldberg R, Haffner S, Ratner R, et al (2005) The effect of metformin and intensive lifestyle intervention on the metabolic syndrome: the Diabetes Prevention Program randomized trial. Ann Intern Med 142: 611–619. 10.7326/0003-4819-142-8-200504190-00009 15838067 PMC2505046

[39] Cauza E, Hanusch-Enserer U, Strasser B, Ludvik B, Metz-Schimmerl S, et al (2005) The relative benefits of endurance and strength training on the metabolic factors and muscle function of people with type 2 diabetes mellitus. Arch Phys Med Rehabil 86: 1527–1533. 10.1016/j.apmr.2005.01.007 16084803

[40] Chen ZP, Stephens TJ, Murthy S, Canny BJ, Hargreaves M, et al (2003) Effect of exercise intensity on skeletal muscle AMPK signaling in humans. Diabetes 52: 2205–2212. 10.2337/diabetes.52.9.2205 12941758

[41] Hardie DG (2011) Sensing of energy and nutrients by AMP-activated protein kinase. Am J Clin Nutr 93: 891S–896. 10.3945/ajcn.110.001925 21325438

[42] Ara I, Larsen S, Stallknecht B, Guerra B, Morales-Alamo D, et al (2011) Normal mitochondrial function and increased fat oxidation capacity in leg and arm muscles in obese humans. Int J Obes (Lond) 35: 99–108. 10.1038/ijo.2010.123 20548301

[43] Steinberg GR, McAinch AJ, Chen MB, O’Brien PE, Dixon JB, et al (2006) The suppressor of cytokine signaling 3 inhibits leptin activation of AMP-kinase in cultured skeletal muscle of obese humans. J Clin Endocrinol Metab 91: 3592–3597. 10.1210/jc.2006-0638 16822822

[44] Calbet JAL (2019) The biological and psychosocial aspects of successful aging in high functional elders: A longitudinal study. Scand J Med Sci Sports 29 Suppl 1: 5–6. 10.1111/sms.13423 31034659

[45] Laursen AH, Kristiansen OP, Marott JL, Schnohr P, Prescott E (2012) Intensity versus duration of physical activity: implications for the metabolic syndrome. A prospective cohort study. BMJ Open 2. 10.1136/bmjopen-2012-001711 23045359 PMC3488727

[46] Stamatakis E, Kelly P, Strain T, Murtagh EM, Ding D, et al (2018) Self-rated walking pace and all-cause, cardiovascular disease and cancer mortality: individual participant pooled analysis of 50 225 walkers from 11 population British cohorts. Br J Sports Med 52: 761–768. 10.1136/bjsports-2017-098677 29858463

[47] Swain DP, Franklin BA (2006) Comparison of cardioprotective benefits of vigorous versus moderate intensity aerobic exercise. Am J Cardiol 97: 141–147. 10.1016/j.amjcard.2005.07.130 16377300

[48] Jefferis BJ, Parsons TJ, Sartini C, Ash S, Lennon LT, et al (2016) Does duration of physical activity bouts matter for adiposity and metabolic syndrome? A cross-sectional study of older British men. Int J Behav Nutr Phys Act 13: 36 10.1186/s12966-016-0361-2 26980183 PMC4793648

[49] Herzig KH, Ahola R, Leppaluoto J, Jokelainen J, Jamsa T, et al (2014) Light physical activity determined by a motion sensor decreases insulin resistance, improves lipid homeostasis and reduces visceral fat in high-risk subjects: PreDiabEx study RCT. Int J Obes (Lond) 38: 1089–1096. 10.1038/ijo.2013.224 24285336 PMC4125749

[50] Dumortier M, Brandou F, Perez-Martin A, Fedou C, Mercier J, et al (2003) Low intensity endurance exercise targeted for lipid oxidation improves body composition and insulin sensitivity in patients with the metabolic syndrome. Diabetes Metab 29: 509–518. 10.1016/S1262-3636(07)70065-4. 14631328

[51] Johnson JL, Slentz CA, Houmard JA, Samsa GP, Duscha BD, et al (2007) Exercise training amount and intensity effects on metabolic syndrome (from Studies of a Targeted Risk Reduction Intervention through Defined Exercise). Am J Cardiol 100: 1759–1766. 10.1016/j.amjcard.2007.07.027 18082522 PMC2190779

[52] Hidalgo-Santamaria M, Fernandez-Montero A, Martinez-Gonzalez MA, Moreno-Galarraga L, Sanchez-Villegas A, et al (2017) Exercise Intensity and Incidence of Metabolic Syndrome: The SUN Project. Am J Prev Med 52: e95–e101. 10.1016/j.amepre.2016.11.021 28082000

[53] Hastert TA, Gong J, Campos H, Baylin A (2015) Physical activity patterns and metabolic syndrome in Costa Rica. Prev Med 70: 39–45. 10.1016/j.ypmed.2014.11.006 25445330 PMC4341893

[54] Sisson SB, Camhi SM, Church TS, Martin CK, Tudor-Locke C, et al (2009) Leisure time sedentary behavior, occupational/domestic physical activity, and metabolic syndrome in U.S. men and women. Metab Syndr Relat Disord 7: 529–536. 10.1089/met.2009.0023 19900152 PMC2796695

[55] Johns DJ, Hartmann-Boyce J, Jebb SA, Aveyard P, Behavioural Weight Management Review G (2014) Diet or exercise interventions vs combined behavioral weight management programs: a systematic review and meta-analysis of direct comparisons. J Acad Nutr Diet 114: 1557–1568. 10.1016/j.jand.2014.07.005 25257365 PMC4180002

[56] Shephard RJ (2003) Limits to the measurement of habitual physical activity by questionnaires. Br J Sports Med 37: 197–206; discussion 206. 10.1136/bjsm.37.3.197 12782543 PMC1724653

[57] Shiroma EJ, Lee IM (2010) Physical activity and cardiovascular health: lessons learned from epidemiological studies across age, gender, and race/ethnicity. Circulation 122: 743–752. 10.1161/CIRCULATIONAHA.109.914721 20713909

[58] Wang Y, Tuomilehto J, Jousilahti P, Antikainen R, Mahonen M, et al (2010) Occupational, commuting, and leisure-time physical activity in relation to heart failure among finnish men and women. J Am Coll Cardiol 56: 1140–1148. 10.1016/j.jacc.2010.05.035 20863955

[59] Chen SP, Chang HC, Hsiao TM, Yeh CJ, Yang HJ (2018) Gender Differences in the Effects of the Frequency of Physical Activity on the Incidence of Metabolic Syndrome: Results from a Middle-Aged Community Cohort in Taiwan. Metab Syndr Relat Disord 16: 224–231. 10.1089/met.2017.0154 29688799

