# Supplementary material for: Domain and intensity of physical activity are associated with metabolic syndrome: A population-based study
Source: PLoS One. 2019 Jul 17;14(7):e0219798. doi: 10.1371/journal.pone.0219798 (PMC6636743; doi:10.1371/journal.pone.0219798)
Supplement: S1 Table — (PDF) [file pone.0219798.s001.pdf]

Appendix A. Physical activities recorded and its distribution by domains and intensities

No.		n	% respon.
**Leisure**		**7554**	**40.9**
**Light-intensity (<3. MET)**		3146	17.1
3	Walking slow (2-3 km/h)	2903	15.7
15	Fishing (standing or sitting)	226	1.2
52	Petanque	9	0.1
55	Pistol shooting	8	0.0
**Moderate (≥3 - ≤ 6MET)**		**3490**	**18.9**
4	Swimming, sea (>150 m)	759	4.1
6	Walking moderate and brisk (>3 km/h)	620	3.4
9	Exercise (gym)	483	2.6
11	Swimming, pool (>150 m)	305	1.7
12	Exercise at home	287	1.6
16	Playing with children	225	1.2
17	Dancing (salon or nightclub)	215	1.2
22	Hunting, small animals	111	0.6
23	Walking, cross-country	101	0.5
24	Aerobic	98	0.5
31	Lifting weights	67	0.4
32	Diving	57	0.3
40	Surfing	23	0.12
42	Basketball recreational	20	0.11
43	Riding	19	0.10
47	Table Tennis	13	0.07
48	Tennis, doubles	12	0.06
49	Sailing (recreational)	10	0.05
50	Fishing, river	10	0.05
51	Golf (shoulder bag)	9	0.05
53	Motorcycling	9	0.05
54	Roller skating	8	0.04
57	Volleyball	7	0.04
62	Scuba diving	4	0.02
64	Rowing, recreational	3	0.02
65	Skiing	3	0.02
67	Golf (pulling/pushing cart)	3	0.02
71	Archery	2	0.01
68	Canoe trip	2	0.01
72	Playing the drums	2	0.01
74	Bowling	1	0.01
73	Water skiing	1	0.01
76	Hunting, large animals	1	0.01
**Vigorous (>6 MET)**		**918**	**5.0**
14	Football	281	1.5
18	Running (8-11 km/h)	185	1.0
25	Cycling (competition)	96	0.5
27	Jogging	82	0.4

No.		n	% respon.
**Leisure (continuation)**			
**Vigorous (continuation)**			
28	Hiking (with back pad)	71	0.4
34	Tennis, singles	47	0.3
35	Martial arts	39	0.2
37	Basketball (competition)	35	0.2
39	Running (12-16 km/h)	25	0.1
41	Squash	22	0.1
44	Climbing	17	0.1
58	Handball	7	0.04
61	Rowing (competition)	4	0.02
66	Skiing	3	0.02
69	Badminton	2	0.01
70	Basketball (referee)	2	0.01
**Domestic**		**5663**	**30.6**
**Light-intensity (<3 MET)**		**4488**	**24.3**
2	Cleaning (house)	3547	19.2
8	Cleaning (garden)	598	3.2
21	Caring, animals	130	0.7
26	Carpentry, indoor	91	0.5
30	Painting, indoor	68	0.4
36	Caring, people	37	0.2
56	Fishing, shellfish	8	0.04
59	Cutting the grass (machine)	5	0.03
63	Miscellaneous	4	0.02
**Moderate (≥3 - ≤ 6 MET)**		**1175**	**6.4**
7	Digging the garden	601	3.3
13	Maintaining and caring, farm	283	1.5
19	Moving furniture	166	0.9
33	Carpentry, outdoor	53	0.3
38	Painting, outdoors	33	0.2
45	Masonry, mechanical	16	0.1
46	Milking	15	0.1
60	Grass/hedge cutting, manual	5	0.03
75	Wood loading	1	0.01
77	Working with hives	1	0.01
78	Shoveling	1	0.01
**Active transport**		**5262**	**28.48**
1	Climbing stairs (>5 floors/day)	4096	22.2
5	Walking (with shopping bags)	622	3.4
10	Walking (home-work)	331	1.8
20	Walking, pulling a cart/trolley	145	0.8
29	Biking to work	68	0.4
**All physical activities**		**18,479**	**100**

nº = positional order in prevalence (n= 78 physical activities); % respon. = % responses, percentage that each PA represents over the total of PAs (n= 18,479)
