# Supplementary material for: Domain and intensity of physical activity are associated with metabolic syndrome: A population-based study
Source: PLoS One. 2019 Jul 17;14(7):e0219798. doi: 10.1371/journal.pone.0219798 (PMC6636743; doi:10.1371/journal.pone.0219798)
Supplement: S2 Table — (PDF) [file pone.0219798.s002.pdf]

Appendix B. Correlations between physical activity domains and intensities, sociodemographic and metabolic syndrome

	Recreat. EE	Domestic EE	Transp. EE	Walking EE	Total EE	Light EE	Modera. EE	Vigorous EE	PA level
Recreational EE	----	-.16 *	-.18 *	.40 *	.57 *	.11 *	.46 *	.18 *	.49 *
Domestic EE		----	.08 *	.09 *	.58 *	.74 *	.13 *	-.12 *	.04 *
Active transport EE			----	.40 *	.09 *	.11 *	-.11 *	.56 *	-.03 *
Total walking				----	.37 *	.43 *	.06 *	.18 *	.06 *
Total EE					----	.58 *	.49 *	.21 *	.49 *
Light EE						----	-.10 *	-.11 *	-.16 *
Moderate EE							----	-.10 *	.76 *
Vigorous EE								----	.17 *
PA level									----
MetS	-.11 *	.10 *	-.07 *	-.00	-.04 *	.10 *	-.04 *	-.15 *	-.05 *
Σ MS components	-.12 *	.12 *	-.07 *	.02	-.03 *	.12 *	-.04 *	-.19 *	-.06 *
TV watching (hours/day)	-.02	.08 *	-.01	.03 *	.03 *	0.1 *	-.01 *	-.06 *	.04 *
Age (groups)	-.15 *	.22 *	-.02	.10 *	.02	.21 *	-.04 *	-.21 *	-.09 *
Sex	.12 *	-.40 *	-.15 *	-.16 *	-.15 *	-.40 *	.12 *	.06 *	.19 *
Educational levels	.14 *	-.23 *	.02	-.03 *	-.05 *	-.19 *	.07 *	.15 *	.11 *
BMI levels	-.09 *	.10 *	-.05 *	.01	-.01	.10 *	-.06 *	-.11 *	-.06 *

* $p < 0.05$ for correlation coefficients; EE= Energy expenditure in MET-h/day; MetS= Metabolic syndrome (binary)
