# Supplementary material for: Domain and intensity of physical activity are associated with metabolic syndrome: A population-based study
Source: PLoS One. 2019 Jul 17;14(7):e0219798. doi: 10.1371/journal.pone.0219798 (PMC6636743; doi:10.1371/journal.pone.0219798)
Supplement: S3 Table — (PDF) [file pone.0219798.s003.pdf]

Appendix C. Odds ratio to have metabolic syndrome in each step of logistic regression analysis

| | | | Bivariate | | PA submodel plus TV | | plus Socio-demographic | | plus Lifestyle | | plus Family history | | plus BMI | |
|---|---|---|---|---|---|---|---|---|---|---|---|---|---|---|
| | | | OR | (95% CI) | OR | (95% CI) | OR | (95% CI) | OR | (95% CI) | OR | (95% CI) | OR | (95% CI) |
| **Physical activity** | Physical Activity | Domain | | | | | | | | | | | | |
| | | Recreational (MET-h/day) | 0.91 | (0.89; 0.92) *** | 0.91 | (0.89; 0.93) *** | 0.93 | (0.91; 0.96) *** | 0.93 | (0.91; 0.96) *** | 0.93 | (0.91; 0.96) *** | 0.95 | (0.93; 0.97) *** |
| | | Domestic (MET-h/day) | 1.09 | (1.07; 1.11) *** | 1.07 | (1.05; 1.09) *** | 1.02 | (0.99; 1.04) | 1.02 | (0.99; 1.04) | 1.02 | (0.99; 1.04) | 1.01 | (0.99; 1.04) |
| | | Active transport (MET-h/day) | 0.90 | (0.84; 0.95) *** | 0.85 | (0.80; 0.91) *** | 0.88 | (0.82; 0.94) *** | 0.88 | (0.82; 0.93) *** | 0.88 | (0.82; 0.94) *** | 0.90 | (0.84; 0.96) ** |
| | | Intensity | | | | | | | | | | | | |
| | | Light (MET-h/day) | 1.08 | (1.06; 1.11) *** | 1.06 | (1.03; 1.08) *** | 1.02 | (1.00; 1.05) | 1.02 | (1.00; 1.05) | 1.02 | (1.00; 1.05) | 1.02 | (0.99; 1.05) |
| | | Moderate (MET-h/day) | 0.93 | (0.91; 0.95) *** | 0.94 | (0.92; 0.96) *** | 0.93 | (0.91; 0.95) *** | 0.93 | (0.91; 0.96) *** | 0.93 | (0.91; 0.96) *** | 0.95 | (0.92; 0.97) *** |
| | | Vigorous (MET-h/day) | 0.83 | (0.80; 0.87) *** | 0.85 | (0.81; 0.89) *** | 0.92 | (0.88; 0.96) *** | 0.91 | (0.88; 0.95) *** | 0.92 | (0.88; 0.95) *** | 0.94 | (0.90; 0.97) *** |
| | | Total PA (MET-h/day) | 0.97 | (0.96; 0.99) *** | 0.97 | (0.96; 0.98) *** | 0.97 | (0.95; 0.98) *** | 0.96 | (0.94; 0.98) *** | 0.97 | (0.95; 0.98) *** | 0.97 | (0.96; 0.99) * |
| | | Total walking (MET-h/day) | 0.99 | (0.96; 1.02) | 0.98 | (0.95; 1.01) | 0.95 | (0.92; 0.98) ** | 0.95 | (0.92; 0.98) ** | 0.95 | (0.92; 0.98) ** | 0.96 | (0.93; 0.99) * |
| | | PA level (≥ 3 MET-h/d vs < 3 MET-h/d) | 0.83 | (0.73; 0.94) ** | 0.82 | (0.72; 0.93) ** | 0.74 | (0.64; 0.85) *** | 0.74 | (0.64; 0.85) *** | 0.74 | (0.64; 0.85) *** | 0.77 | (0.66; 0.91) *** |
| **Socio-demographic** | Age | 18-30 | 1.00 | | | | 1.00 | | 1.00 | | 1.00 | | 1.00 | |
| | | 31-45 | 3.26 | (2.6; 4.1) *** | | | 2.77 | (2.20; 3.50) *** | 2.75 | (2.18; 3.47) *** | 2.56 | (2.02; 3.23) *** | 2.17 | (1.68; 2.79) *** |
| | | 46-60 | 8.78 | (7.0; 11.0) *** | | | 6.41 | (5.05; 8.14) *** | 5.94 | (4.67; 7.55) *** | 5.59 | (4.39; 7.12) *** | 3.81 | (2.94; 4.95) *** |
| | | > 60 | 16.3 | (12.4; 21.4) *** | | | 10.34 | (7.76; 13.78) *** | 9.08 | (6.78; 12.15) *** | 8.57 | (6.39; 11.49) *** | 6.63 | (4.82; 9.12) *** |
| | Gender (men *vs.* women) | | 1.18 | (1.05; 1.32) ** | | | 1.49 | (1.29; 1.72) *** | 1.48 | (1.28; 1.72) *** | 1.52 | (1.31; 1.77) *** | 1.42 | (1.20; 1.67) *** |
| | Education | University | 1.00 | | | | 1.00 | | 1.00 | | 1.00 | | 1.00 | |
| | | Secondary | 1.40 | (1.13; 1.75) *** | | | 1.51 | (1.19; 1.90) *** | 1.51 | (1.20; 1.92) *** | 1.50 | (1.18; 1.90) *** | 1.20 | (0.93; 1.56) |
| | | Primary or lesser | 3.59 | (2.94; 4.38) *** | | | 2.06 | (1.65; 2.56) *** | 2.06 | (1.65; 2.57) *** | 2.02 | (1.62; 2.52) *** | 1.38 | (1.09; 1.76) *** |
| | Ancestry (yes *vs* . no) | | 1.25 | (1.07; 1.45) ** | | | 1.05 | (0.89; 1.25) | 1.03 | (0.87; 1.22) | 1.02 | (0.86; 1.21) | 1.06 | (0.88; 1.27) |
| | Housekeeper (yes vs. no) | | 2.26 | (1.98; 2.59) *** | | | 1.72 | (1.45; 2.05) *** | 1.7 | (1.43; 2.02) *** | 1.71 | (1.44; 2.04) *** | 1.59 | (1.31; 1.93) *** |
| **Lifestyle** | TV watching | < 1 h/day | 1.00 | | 1.00 | | 1.00 | | 1.00 | | 1.00 | | 1.00 | |
| | | 1 - < 2 h/day | 0.97 | (0.84; 1.12) | 0.97 | (0.84; 1.11) | 0.94 | (0.81; 1.09) | 0.95 | (0.82; 1.11) | 0.94 | (0.81; 1.10) | 0.93 | (0.79; 1.10) |
| | | 2 - < 3 h/day | 1.22 | (1.05; 1.41) ** | 1.23 | (1.06; 1.43) *** | 1.09 | (0.93; 1.28) | 1.10 | (0.94; 1.29) | 1.09 | (0.92; 1.28) | 1.09 | (0.91; 1.30) |
| | | 3 - < 4 h/day | 1.92 | (1.52; 2.42) *** | 1.93 | (1.53; 2.44) *** | 1.51 | (1.17; 1.94) *** | 1.52 | (1.18; 1.96) *** | 1.52 | (1.18; 1.96) *** | 1.35 | (1.03; 1.78) * |
| | | > 4 h/day | 2.87 | (1.99; 4.13) *** | 2.90 | (2.01; 4.16) *** | 1.76 | (1.18; 2.63) *** | 1.80 | (1.20; 2.69) *** | 1.73 | (1.15; 2.58) ** | 1.64 | (1.06; 2.54) * |
| | Night-time sleep (< 6 vs. ≥ 6 h/day) | | 1.63 | (1.45; 1.83) *** | | | | | 1.37 | (1.16; 1.61) *** | 1.37 | (1.16; 1.61) *** | 1.24 | (1.03; 1.49) * |
| | Smoker (yes *vs* . no) | | 0.67 | (0.59; 0.77) *** | | | | | 0.76 | (0.66; 0.88) ** | 0.75 | (0.65; 0.87) ** | 0.97 | (0.82; 1.14) |
| | Alcohol intake (high vs low) | | 1.40 | (1.12; 1.76) *** | | | | | 1.17 | (0.89; 1.54) | 1.15 | (0.87; 1.51) | 1.09 | (0.81; 1.47) |
| | Nap (yes *vs* . no) | | 1.45 | (1.28; 1.64) *** | | | | | 1.15 | (1.00; 1.32) * | 1.16 | (1.01; 1.33) * | 1.18 | (1.01; 1.37) * |
| **Family history** | Family history of CVD and diabetes | Neither | 1.00 | | | | | | | | 1.00 | | 1.00 | |
| | | Paternal | 1.2 | (1.02; 1.43) * | | | | | | | 1.26 | (1.04; 1.51) * | 1.27 | (1.03; 1.55) * |
| | | Maternal | 1.55 | (1.32; 1.81) *** | | | | | | | 1.38 | (1.16; 1.64) *** | 1.40 | (1.16; 1.69) *** |
| | | Both parents | 1.89 | (1.62; 2.21) *** | | | | | | | 1.63 | (1.37; 1.92) *** | 1.58 | (1.31; 1.90) *** |
| **Obesity** | BMI | Normal | 1.00 | | | | | | | | | | 1.00 | |
| | | Overweight | 8.4 | (6.8; 10.4) *** | | | | | | | | | 5.9 | (4.7; 7.3) *** |
| | | Obese | 30.9 | (24.8; 38.6) *** | | | | | | | | | 21.1 | (16.8; 26.6) *** |
